# Malaria Diagnosis at the Pediatric Emergency Unit of a Teaching Hospital in Makurdi, North Central Nigeria

**DOI:** 10.4314/ejhs.v34i1.5

**Published:** 2024-01

**Authors:** A Michael, NB Samba, MG Adikwu, MO Ochoga, JU Akpan, EE Eseigbe

**Affiliations:** 1 Department of Pediatrics, Benue State University/ Benue State University Teaching Hospital, Makurdi; 2 Department of Pediatrics, Benue State University Teaching Hospital, Makurdi; 3 Department of Pediatrics, Federal University of Health Sciences, Otukpo; 4 Department of Pediatrics, Federal Medical Center, Makurdi

**Keywords:** Malaria, emergency unit, hospital, over-diagnosis

## Abstract

**Background:**

Globally, there were 241 million cases of malaria in 2020, with an estimated 627,000 deaths with Nigeria accounting for 27% of the global malaria cases. In sub-Saharan Africa, testing is low with only 28% of children with a fever receiving medical advice or a rapid diagnostic test in 2021. In Nigeria, there are documented reports of over-diagnosis and over-treatment of malaria in children. Therefore, this study examined the diagnosis of malaria at the Benue State University Teaching Hospital, Makurdi.

**Methods:**

A 5-year (2018-2022) retrospective study was carried out at the Emergency Pediatric Unit (EPU). Records of all children presenting to the EPU with an assessment of malaria were retrieved and reviewed. Data was analyzed using SPSS 23.

**Results:**

Out of 206 children reviewed, 128 (62.1%) were tested using either malaria RDT or microscopy while 78(37.9%) were not tested. Out of the number tested, 59(46.1%) were negative while 69(53.9%) tested positive, of which 14(20.3%) had uncomplicated malaria while 55(79.7%) had severe malaria. However, while 97.1% (n=67) of the positive cases were treated with IV artesunate, 69.5% (n=41) of those who tested negative and 88.5% (69) of those who were not tested also received IV artesunate. Moreover, while 85.5% (n=59) of those who tested positive received oral artemisinin-based combination therapy (ACT), 72.9% (n=43) of those who tested negative and 67.9% (53) of those who were not tested also received oral ACT.

**Conclusion:**

There was over-diagnosis of malaria, and subsequently, over-treatment. Hence continued emphasis on parasitological confirmation of malaria before treatment is recommended.

## Introduction

Malaria is an acute febrile illness caused by plasmodium parasites which are spread through the bites of infected female anopheles mosquitoes (1). *P.falciparum* is the deadliest malaria parasite and the most prevalent on the African continent, and it accounts for about 97% of uncomplicated malaria and is also the species most responsible for the severe form of the disease that leads to death in Nigeria (1,2). Malaria is characterized by recurrent symptoms of fever, chills and generalized body pain^(2)^. The first symptoms, fever, headache and chills usually appear 10-15 days after the infective mosquito bite and may be mild and difficult to recognize as malaria (1). However, when left untreated, *P. falciparum* malaria can progress to severe illness and death within a period of 24 hours (1,2). The disease occurs in all age groups, and though severe disease is seen in young children and pregnant women, malaria prevalence is highest in children under 5 years of age (2,3).

Globally, there were 241 million cases of malaria in 2020, with an estimated 627,000 deaths(1,4). Nigeria accounted for 27% of the global malaria cases and 31% of the global malaria deaths in 2020 (1,4). There are 63 million clinical cases of malaria annually in Nigeria with 90,000 deaths annually, and malaria is the leading cause of death in children aged 1-59 months in Nigeria (4,5). Every 75 seconds, a child under 5 years dies of malaria and many of these deaths are preventable and treatable hence malaria is an urgent public health priority (6). The disease and the costs of its treatment trap families in a cycle of illness, suffering and poverty (6). Today, nearly half of the world's population, most of whom live in sub-Saharan Africa are at risk of developing malaria and facing its economic challenges (6). Malaria transmission is stable in Nigeria but with high seasonal variation in the northern half of the country, and while the transmission season can last all year round in the south, it is about 3 months or less in the northern part of the country (7,8). The incidence of malaria in Benue was reported to be 13% while a previous survey reported the prevalence of 32.3% for *P.falciparum* parasitemia among children aged 0-12 months and 50% for children aged 49-60 months in Makurdi (9,10).

Whilst fever is an indicator of malaria in children, it can be a sign of other acute infections (8). Hence the WHO recommends the universal use of diagnostics testing to confirm malaria infection before administering any treatment(6,8,11). . Malaria is diagnosed in febrile children by a rapid diagnostic test (RDT) which involves taking blood sample from the finger or heel to test for malaria plasmodium (P) antigens(6,8,11). Diagnosis can also be made via malaria microscopy which is considered as the Gold standard in malaria diagnosis; however, this is subject to the skill and experience of the microscopist (8). In sub-Saharan Africa, testing is low with only 28% of children with a fever receiving medical advice or a rapid diagnostic test in 2021 and in Nigeria. There are documented reports of over-diagnosis without parasitological confirmation and over-treatment of malaria in children (6,12). According to the 2021, National Malaria Indicator Survey, among children under age 5 years of age with fever in the 2 weeks preceding the survey, only 24.3% had blood taken from a finger or heel for testing (13). Diagnostic testing enables health workers to swiftly distinguish between malarial and non-malarial fevers, facilitating appropriate treatment with artemisinin-based combination therapy (ACT) (1,8).

Early diagnosis and treatment of malaria reduces disease, prevents deaths and contributes to reducing malaria transmission (8) In line with the WHO recommendation, the country has adopted the test, treat and track strategy to ensure that all suspected cases of malaria are properly diagnosed using rapid diagnostic tests or microscopy, treated promptly with recommended ACT where the test result is positive and documented (2,8,11,14).

Hence, this study sought to determine the rate of testing of malaria as well as evaluate the appropriateness of treatment given to children seen at the emergency unit of the Department of Pediatrics, Benue State University Teaching Hospital Makurdi.

## Material and Methods

This was a 5-year (January 2018- September 2022) retrospective study carried out at the Emergency Pediatric Unit (EPU) of the Department of Pediatrics, Benue State University Teaching Hospital, Makurdi. The Benue State University Teaching Hospital is located in Makurdi which is the capital of the State. It provides general care and specialist services for patients within the state and its surrounding communities as well as serving as a referral center. The emergency unit of the Department of Pediatrics attends to children presenting to the hospital from home, as well as following referrals from other hospitals and those referred from the General Outpatient Department (GOPD) of the hospital. The unit is manned by a consultant and Pediatric residents in conjunction with nurses. As a unit protocol, children presenting with symptoms suggestive of malaria undergo a rapid diagnostic test (RDT for *P.falciparum*) in the EPU(8).^(^. Subsequently, blood samples were collected and sent to the laboratory which was manned by a trained malaria microscopist for microscopy. Children seen in the emergency unit were those between the ages of 1 month and 15 years. Records of all children admitted into the unit with history of fever (axillary temperature ≥37.5°c), chills, headache, pallor, splenomegaly, etc and with an assessment of either uncomplicated or severe malaria during the period under review were retrieved and assessed using the study proforma^(8)^. Uncomplicated malaria was defined as symptomatic infection with malaria parasitemia without signs of severity and/or evidence of vital organ dysfunction (8). Severe malaria was defined as acute falciparum malaria with signs of severity and/or evidence of vital organ dysfunction (8). The socio-demographic details of the children including age, sex, address and other details including use of long-lasting insecticide treated nets (LLINs) were documented in the study proforma. Socio-economic classification was based on the Ogunlesi classification (15). Data was analyzed using IBM SPSS Statistic software version 23. Chi-squared test and Fisher's exact test were used as appropriate. The level of significance was set at P < 0.05. Ethical clearance was obtained from the BSUTH Health Research Ethics Committee.

## Results

Total admission over the period under review was 1960, out of which 206 children had an assessment of malaria and were reviewed for which 3.5% had confirmed malaria. The majority of the children were 5 years of age and below, with male-to-female ratio of 1.1:1 and were mostly from social class III-V as shown in Table 1.

**Table 1:** Socio-demographic characteristics

Variables	Frequency	Percent
Age		
<1	20	9.7
1-2	66	32.0
3-5	66	32.0
6-8	27	13.1
9-11	12	5.8
12-15	15	7.3
Sex		
Male	108	52.4
Female	98	47.6
Religion		
Christianity	200	97.1
Muslim	6	2.9
Tribe		
Tiv	160	77.7
Idoma	21	10.2
Igbo	11	5.3
Others	14	6.8
Social Class		
Social Class III	39	18.9
Social Class IV	34	16.5
Social Class V	61	29.6

Out of 128(62.1%) children who were tested using malaria RDT or Microscopy, 69 (33.5%) were positive, while 59 (28.6%) were negative.

Notably 77 (38.5%) of the patients who were managed as malaria had no test done even though they presented with a fever, while 33% of those presenting with a fever tested positive as shown in Table 2. A significant number of patients seen were not tested using either RDT (54.9%) or microscopy (67%), and most of them had no test done for random blood glucose (71.4%) as seen in Table 3.

**Table 2:** Clinical presentation and malaria test cross tabulation

Variable		Malaria			Test statistics	p-value

		Positive n(%)	Negative n(%)	Not done n(%)		
Fever					Fisher's exact=1.40	0.586
	Yes	66(33.0)	57(28.5)	77(38.5)		
	No	3(50.0)	2(33.3)	1(16.7)		
Chills					χ^2^=2.41	0.299
	Yes	15(39.5)	7(18.4)	16(42.1)		
	No	54(32.1)	52(31.0)	62(36.9)		
Headache					χ^2^=0.81	0.666
	Yes	16(39.0)	10(24.4)	15(36.6)		
	No	53(32.1)	49(29.7)	63(38.2)		
Generalized Body Weakness					χ^2^=0.75	0.687
	Yes	18(36.7)	15(30.6)	16(32.7)		
	No	51(32.5)	44(28.0)	62(39.5)		
Joint Paint					χ^2^=0.84	0.655
	Yes	3(50.0)	1(16.7)	2(33.3)		
	No	66(33.0)	58(29.0)	76(38.0)		
Vomiting					χ^2^=13.02	0.001
	Yes	33(29.2)	44(38.9)	36(31.9)		
	No	36(38.7)	15(16.1)	42(45.2)		
Diarrhea					χ^2^= 10.46	0.005
	Yes	15(24.6)	27(44.3)	19(31.1)		
	No	54(37.2)	32(22.1)	59(40.7)		
Convulsions					χ^2^=3.75	0.153
	Yes	32(36.4)	19(21.6)	37(42.0)		
	No	37(31.4)	40(33.9)	41(34.7)		
Jaundice					Fisher's exact=8.27	0.011
	Yes	9(69.2)	3(23.1)	1(7.7)		
	No	60(31.1)	56(29.0)	77(39.9)		
Reduced Urine Output					Fisher's exact=2.11	0.384
	Yes	1(20.0)	3(60.0)	1(20.0)		
	No	68(33.8)	56(27.9)	77(38.3)		
Fast/difficulty breathing					χ^2^=3.47	0.176
	Yes	17(44.7)	7(18.4)	14(36.8)		
	No	52(31.0)	52(31.0)	64(38.1)		
Loss of consciousness					χ^2^=5.23	0.073
	Yes	19(38.0)	8(16.0)	23(46.0)		
	No	50(32.1)	51(32.7)	55(35.3)		

**Table 3:** Investigation results for presumed malaria cases

Variables	Frequency	%
RDT for Malaria		
Positive	57	27.7
Negative	36	17.5
Not done	113	54.9
Blood film for malaria parasite (MP)		
MP Seen	25	12.1
MP Not seen	43	20.9
Not done	138	67.0
Full Blood Count		
Anemia	98	47.6
Leucopenia	1	.5
Thrombocytopenia	8	3.9
Anemia + Thrombocytopenia	14	6.8
Anemia + Leucopenia +	1	0.5
Thrombocytopenia		
None	84	40.8
Random Blood Glucose		
Low	22	10.7
Normal	37	18.0
Not done	147	71.4

Testing rate was 62.1%, as 128 patients were tested with 33 patients having both RDT and microscopy done, while 78 had neither RDT nor Microscopy done. A total of 60 patients had RDT done but no microscopy while 35 patients had microscopy done without RDT as shown in Table 4.

**Table 4:** Blood film for malaria parasite (MP) and RDT for malaria Cross-tabulation

Blood film for malaria parasite			RDT for malaria			Total

			Positive	Negative	Not done	
Blood film for malaria parasite (MP)	MP Seen	Count	13	6	6	25
		% within Blood film for malaria parasite (MP)	52.0%	24.0%	24.0%	100.0%
		% within RDT for malaria	22.8%	16.7%	5.3%	12.1%
	MP Not seen	Count	5	9	29	43
		% within Blood film for malaria parasite (MP)	11.6%	20.9%	67.4%	100.0%
		% within RDT for malaria	8.8%	25.0%	25.7%	20.9%
	Not done	Count	39	21	78	138
		% within Blood film for malaria parasite (MP)	28.3%	15.2%	56.5%	100.0%
		% within RDT for malaria	68.4%	58.3%	69.0%	67.0%
Total		Count	57	36	113	206
		% within Blood film for malaria parasite (MP)	27.7%	17.5%	54.9%	100.0%
		% within RDT for malaria	100.0%	100.0%	100.0%	100.0%

Most patients seen were treated with intravenous artesunate (85.9%) and oral ACT (75.2%), and artemether-lumefantrine was the most prescribed oral ACT (98.7%) as shown in Table 5.

**Table 5:** Treatment for Malaria cases

Variables		Frequency	Percent
IV Artesunate			
	Yes	177	85.9
	No	29	14.1
Oral ACT			
	Yes	155	75.2
	No	51	24.8
Artemether-Lumefantrine (n=155)			
	Yes	153	98.7
	No	2	1.3
Artesunate/Amodiaquine (n=155)			
	Yes	2	1.3
	No	153	98.7
Blood transfusion			
	Yes	80	38.8
	No	126	61.2

Both those who tested either positive or negative for malaria were treated. Even those who were not tested were treated with IV artesunate and subsequently oral ACT as shown in Table 6.

**Table 6:** and Treatment of malaria cross-tabulation

Variable		Malaria			Test statistics	p-value

		Positive n (%)	Negative n (%)	Not done n (%)		
IV Artesunate					χ^2^=20.71	0.000
	Yes	67(37.9)	41(23.2)	69(39.0)		
	No	2(6.9)	18(62.1)	9 (31.0)		
Oral ACT					χ^2^=6.30	0.043
	Yes	59(38.1)	43(27.7)	53(34.2)		
	No	10(19.6)	16(31.4)	25(49.0)		
Artemether-Lumefantrine (n=155)						
	Yes	58(37.9)	42(27.5)	53(34.6)	-	-
	No	0(0.0)	0(0.0)	0(0.0)		
Artesunate/Amodiaquine (n=2)						
	Yes	1(50.0)	1(50.0)	0(0.0)	-	-
	No	0(0.0)	0(0.0)	0(0.0)		
Blood transfusion					χ^2^=5.21	0.074
	Yes	32(40.0)	16(20.0)	32(40.0)		
	No	37(29.4)	43(34.1)	46(36.5)		

## Discussion

Malaria still remains one of the leading causes of under 5 morbidity and mortality in Nigeria (5). The results of this study showed that the majority of the cases of malaria seen were in children aged 5 years and below in keeping with other reports such as Jombo *et al* in Makurdi (10) and Ahmad *et al* in Sokoto (3).

Most of the children for whom an assessment of malaria was made presented with fever (33%), and this is in keeping with the report of Oladosu *et al* (12), in Lagos, but in contrast with the report of Jombo *et al* who reported fever in only 1.8% of the children (10). However, 28.5% of the children presenting with fever tested negative for malaria which is similar to the report of Oleribe *et al* in Abuja (16), and this underscores the need to test every patient presenting with fever before treatment as not every fever in children is due to malaria (17).

Even though Nigeria has adopted the test, treat and track strategy (3T) with the aim to ensure that all suspected cases of malaria are diagnosed with either rapid diagnostic tests or microscopy (8), only 62.1% of the children who had an assessment of malaria received a parasitological test either with an RDT or microscopy. Interestingly, 37.9% of the patients for whom an assessment of malaria was made were not tested. This leads to over-diagnosis of malaria as even those who were not tested are given an assessment of malaria. This could mask other causes of fever in the children and may delay appropriate diagnosis of the underlying cause and can affect the outcome as attention is not paid to other causes of fever in the child (18,19).

Among those who were tested, there was double testing with both RDT and microscopy. This is contrary to the National Guidelines for diagnosis and treatment of malaria which stipulates that all suspected malaria cases should have a prompt parasitological confirmatory test using either microscopy or RDTs (8). The use of the RDTs for parasitological diagnosis is a key recommendation of the National Guidelines even as the RDTs in use in the country have been shown to have sensitivity of ≥ 95% (2,8,20). This practice of double testing may be due to a reluctance to change as it is seen to be a screening test amongst some clinicians or even lack of confidence in the RDTs as reported by Boadu *et al* (21). Moreover, both those who tested positive and those who tested negative as well as those who were not tested received intravenous artesunate, and subsequently, an Oral ACT. This clearly showed over-treatment of the children seen at the emergency unit, and this is similar to the report of Oladosu in Lagos (12), Onchiri *et al* in Kenya(22).^)^, Manguin *et al* in Angola (23) and Peterson *et al* in Malawi (24), who also reported treatment of malaria despite negative laboratory tests. Over-treatment of malaria leads to undue consumption of antimalarials which has cost implication for the economy, and it could also lead to unnecessary adverse drug reactions and development of resistance to antimalarials as well as affect the quality of data on malaria diagnosis (17,23). According to the world malaria report, Nigeria distributed 17.9 million ACTs in 2020, and it is likely that over-diagnosis and over-treatment could affect the numbers of ACTs consumed for treating non-malaria fevers (4). The most commonly prescribed oral ACT in this study was artemether-lumefantrine which is the first line drug for uncomplicated malaria according to the National Guidelines and was prescribed in 98.7% of patients given oral ACTs. This is in keeping with the report of Mbah *et al* (25) in Calabar which documented a high rate ACT prescription by clinicians but in contrast to Welle *et al*'s (26) study which reported low preference for ACT among health workers in Lokoja, Kogi State. This pattern of prescription may be due to the level of awareness of the oral ACTS, or lack of confidence in the other drugs on the National Guideline. In conclusion, most of the children for whom an assessment of malaria was made were 5 years and below which underscores the need for continued focus on this age group. However, there was over diagnosis of malaria and subsequently over-treatment which leads to unwarranted use of antimalarial drugs. Hence, continued emphasis should be placed on parasitological confirmation of malaria before treatment, continued engagement of health workers on malaria case management and continued awareness about the role of non-malaria fevers in children in malaria endemic regions.

## Figures and Tables

**Figure 1:**
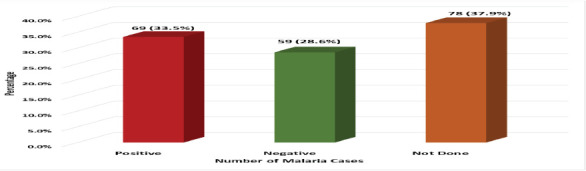
Number of presumed malaria cases
